# Augmented antitumor activity by olaparib plus AZD1775 in gastric cancer through disrupting DNA damage repair pathways and DNA damage checkpoint

**DOI:** 10.1186/s13046-018-0790-7

**Published:** 2018-06-28

**Authors:** Xiaoting Lin, Dongshao Chen, Cheng Zhang, Xiaotian Zhang, Zhongwu Li, Bin Dong, Jing Gao, Lin Shen

**Affiliations:** 1 Department of Gastrointestinal Oncology, Key Laboratory of Carcinogenesis and Translational Research (Ministry of Education/Beijing), Peking University Cancer Hospital and Institute, Fu-Cheng Road 52, Hai-Dian District, Beijing, 100142 China; 2 Department of Pathology, Key Laboratory of Carcinogenesis and Translational Research (Ministry of Education/Beijing), Peking University Cancer Hospital and Institute, Beijing, 100142 China

**Keywords:** PARP inhibitor, WEE1/PLK1 dual inhibitor, Combination, Gastric cancer, HR deficiency, DNA damage checkpoint

## Abstract

**Background:**

Targeting poly ADP-ribose polymerase (PARP) has been recently identified as a promising option against gastric cancer (GC). However, PARP inhibitors alone achieve limited efficacy. Combination strategies, especially with homologous recombination (HR) impairment, are of great hope to optimize PARP inhibitor’s efficacy and expand target populations but remains largely unknown. Herein, we investigated whether a WEE1/ Polo-like kinase 1 (PLK1) dual inhibitor AZD1775 reported to impair HR augmented anticancer activity of a PARP inhibitor olaparib and its underlying mechanisms.

**Methods:**

GC cell lines and in vivo xenografts were employed to determine antitumor activity of PARP inhibitor combined with WEE1/PLK1 dual inhibitor AZD1775. Western blot, genetic knockdown by siRNA, flow cytometry, Immunohistochemistry were performed to explore the underlying mechanisms.

**Results:**

AZD1775 dually targeting WEE1/PLK1 enhanced effects of olaparib on growth inhibition and apoptotic induction in GC cells. Mechanistic investigations elucidate that WEE1/PLK1 blockade downregulated several HR-related proteins and caused an accumulation in γH2AX. As confirmed in both GC cell lines and mice bearing GC xenografts, these effects were enhanced by AZD1775-olaparib combination compared to olaparib alone, suggesting that disrupting HR-mediated DNA damage repairs (DDR) by WEE1/PLK1 blockade might be responsible for improved GC cells’ response to PARP inhibitors. Given the DNA damage checkpoint as a primary target of WEE1 inhibition, our data also demonstrate that AZD1775 abrogated olaparib-activated DNA damage checkpoint through CDC2 de-phosphorylation, followed by mitotic progression with unrepaired DNA damage (marked by increased pHH3-stained and γH2AX-stained cells, respectively).

**Conclusions:**

PARP inhibitor olaparib combined with WEE1/PLK1 dual inhibitor AZD1775 elicited potentiated anticancer activity through disrupting DDR signaling and the DNA damage checkpoint. It sheds light on the combination strategy of WEE1/PLK1 dual inhibitors with PARP inhibitors in the treatment of GC, even in HR-proficient patients.

**Electronic supplementary material:**

The online version of this article (10.1186/s13046-018-0790-7) contains supplementary material, which is available to authorized users.

## Background

Gastric cancer (GC) is one of the most common malignancies and a leading cause of cancer-related mortality in China [1]. Although emerging targeted strategies have brought new hope to antitumor therapy, options for advanced GC with high heterogeneity are still few, only three drugs (trastuzumab, ramucirumab and apatinib) have been currently approved, and the prognosis of advanced GC remains poor. Hence, development of novel strategies against advanced GC is urgently needed.

Poly ADP-ribose polymerase (PARP) inhibitors that competitively combine and trap PARP to disrupt (SSB) single-strand DNA breaks repairs and elicit anticancer activity emerge as a promising strategy for GC [2–4]. However, PARP inhibitors alone exert limited efficacy in the treatment of cancers and how to optimize PARP inhibitors’ eligible populations and effectiveness remain poorly understood. Of interest, SSB can be converted into double-strand DNA breaks (DSB), which results in treatment failure of targeting PARP when homologous recombination (HR) is functional [2, 3]. Thus, defects in HR has been identified as a predictor for PARP inhibitors’ sensitivity. For instance, PARP inhibitors olaparib and rucaparib have been approved to treat BRCA-defective ovarian or prostate cancer patients [5] while GC patients harboring low-ATM gains greater survival benefit than high-ATM patients when treated with olaparib plus paclitaxel [4]. Cancers deficient in alternative HR-related factors like RAD51, 53BP1, ARID1A and CCDC6 are also proved sensitive to PARP inhibitors [3, 6, 7]. Based on these, compromising HR functions has been proposed to improve PARP inhibitors’ efficacy against cancers and even expand uses of PARP inhibitors to a greater population with functional HR [8–13]. However, whether HR deficiency inducers enhance responses of GC to PARP inhibitors and its underlying mechanisms remain uninvestigated, which largely restricts the use of PARP inhibitors.

WEE1 kinase is a gatekeeper of the DNA damage checkpoint (a.k.a. G_2_/M checkpoint) that allows DNA repair before mitotic entry [14]. As validated in preclinical models, WEE1 suppression is an emerging strategy against GC [15]. Of note, targeting WEE1 results in HR defects [16–19], suggesting WEE1 blockade as a promising option for PARP inhibitor-contained combination strategies. However, apart from an ongoing phase Ib study addressing WEE1 inhibitor AZD1775 combined with olaparib against refractory solid tumors (www.clinicaltrials.gov; NCT02511795), therapeutic potentials of PARP/WEE1 dual blockade and its effect on HR impairment against cancer remain to be revealed. Moreover, the widely-used WEE1 inhibitor AZD1775 also targets Polo-like kinase 1 (PLK1) that has been reported to impact on PARP inhibitor’s efficacy [20, 21]. Nevertheless, whether PLK1 inhibition by AZD1775 play a critical role in effectiveness of AZD1775-PARP inhibitor combination is elusive.

In this work, we investigated the therapeutic potential and underlying mechanisms of a PARP inhibitor olaparib plus a WEE1/PLK1 dual inhibitor AZD1775 in GC cell lines and preclinical models. Our study sheds light upon the improvement of current PARP targeted therapy and provides evidence for further clinical investigation.

## Materials and Methods

### Reagents and antibodies

AZD1775 and olaparib were purchased from Selleck Chemicals (Houston, TX). Reagents were formulated and stored according to manufacturer’s protocols for in vitro and in vivo experiments. Following antibodies were used: primary antibodies against caspase 3 (#9664), Bax (#5023), MRE11 (#4847), NBS1 (#14956), ATM (#2873), RAD51 (#8875), 53BP1 (#4937), γH2AX (#9718), cyclinB1 (#12231), CDC2 (#28439), pCDC2 (Y15; #4539), pHH3 (#53348), ATR (#2790), pATR (S428; #2853),Chk1 (#2360), pChk1 (S317; #12302), pChk1(S345, #2348), histone H3 (#4499) and secondary HRP-conjugated goat anti-rabbit (#7074) and anti-mouse antibodies (#7076) from Cell Signal Technology (CST, Danvers, MA); anti-RPA32 (#ab76420) from Abcam (Cambridge, MA); anti-pRPA32 (S4/S8, #A300-245A) and anti-pRPA32 (S33, #A300-246A) from Bethyl Laboratories (Montgomery, TX); anti-PARP1(#Sc-8007) from Santa Cruz (Dallas, TX); anti-PAR (Ab-1, 10H; #AM80) from Merck Millipore, (Darmstadt, Germany); and anti-β-actin (#A5441) from Sigma-Aldrich (St. Louis, MO).

### Cell lines and cell culture

Human GC cell lines MKN45 and AGS were kindly provided by Professor Youyong Lv (Peking University Cancer Hospital & Institute). Cell lines were cultured in RPMI 1640 medium (Gibco BRL, Gaithersburg, MD) supplemented with 10% fetal bovine serum (Gibco BRL), 1% penicillin and streptomycin (HyClone, Logan, UT), and incubated in a humidified incubator (37 °C) with 5% CO_2_.

### Cell viability assays

Cells (4000 cells/well) were seeded in 96-well plates and allowed to adhere overnight in complete medium. Following drug treatment, cell viability was measured using a CCK-8 commercial kit (Dojindo laboratories, Tokyo, Japan) according to the manufacturer’s protocol. Absorbance was measured at 450 nm using a spectrophotometer.

Combination effects were evaluated after exposure to either AZD1775 alone or in combination with olaparib at a fixed concentration ratio of 1:200 (AZD1775: olaparib) using combination index (CI). The CI values were calculated with CompuSyn Version 1.0 software (ComboSyn Inc., Paramus, NJ) by Chou-Talalay equation [22]: CI = (D)_1_ /(D_χ_)_1_ + (D)_2_ /(D_χ_)_2_. In this equation, (D_χ_)_1_ and (D_χ_)_2_ represented concentrations of each drug alone to exert χ% effect while (D)_1_ and (D)_2_ were concentrations of drugs in combination to elicit the same effect. CI < 1, = 1 and > 1 indicated synergism, additivity and antagonism, respectively. Fraction affected (F_a_) represented fraction affected by a given drug concentration and evolution of drug interactions was assessed by F_a_-CI plot.

### Flow cytometry

Cells were treated with 0.3 μM AZD1775 in the presence or absence of 20 μM olaparib. For apoptosis analysis, cells were collected, washed in PBS and double-stained using an Annexin V-Phycoerythrin (PE)/7-amino-actinomycin (7-AAD) apoptosis detection kit (BD Biosciences, Erembodegem, Belgium) following the vendor’s protocol. For cell cycle analysis, cells were digested with trypsin, washed in PBS, fixed in 70% immediately prepared precooled ethanol overnight at 4 °C. After PBS washing, cells were stained with a propidium iodide (PI)/RNase staining buffer (BD Biosciences) at room temperature for 15 min in the dark according to the manufacturer’s instruction. For quantifying pHH3 and γH2AX positive cells at separate cell cycle stages, cells were fixed in 70% ice-cold ethanol overnight at 4 °C, permeabilized with 0.5% Triton X-100 (Amresco, Solon, OH) for 10 min at room temperature, incubated with primary antibodies against pHH3 (1:300) and γH2AX (1:200), probed with the FITC-conjugated goat anti-rabbit antibody (1:200; ZSGB-BIO, Beijing, China) and stained with a PI/RNase staining buffer (BD Biosciences) as described in protocols. All steps were followed by PBS washing. Samples were detected by flow cytometry within 1 h (BD Biosciences) and analyzed using FlowJo Version 7.6.1 software (FlowJo, Ashland, OR) or ModFit Version 3.0 software (Verity Software House, Topsham, ME).

### Immunoblotting analysis

After drug treatment, GC cells and tumor tissues were lysed using a CytoBuster protein extraction reagent (Merck Millipore) in the presence of protease and phosphatase inhibitor cocktail tablets (Roche, Basle, Switzerland). Chromatin fractions were extracted as previously described [23]. Protein concentration was measured with a BCA protein assay Kit (Beyotime, Jiangsu, China). Soluble lysates were subjected to SDS-PAGE and transferred to PVDF membranes (Merck Millipore). After blocking with 5% BSA (Amresco) or fat-free milk, membranes were probed with primary antibodies (1:1000 diluted in blocking solutions except 1:10,000 for β-actin) at 4 °C overnight and secondary antibodies (1:2000) at room temperature for 1 h. Signals were visualized using Amersham Imager 600 (GE Healthcare, Chicago, IL) after incubation with Clarity Western ECL substrate (Bio-Rad, Hercules, CA). Trapped/total PARP1 was quantified by Image J Version 1.48 software (NIH, Bethesda, MD).

### Genetic knockdown by siRNA

a WEE1 siRNA kit was purchased from RiboBio (Guangzhou, China). PLK1 siRNA (#6292) and negative control siRNA (#6568) were purchased from CST. Cells were seeded in 6-well plates and transfected at about 80% confluence with WEE1/PLK1 siRNAs and their corresponding negative control siRNA by Lipofectamine 3000 (Invitrogen, Carlsbad, CA) according to manufacturer’s protocol. Cells transfected for 48 h were harvested for immunoblotting analysis.

### In vivo studies

MKN45 cells were detached with trypsin/EDTA (Gibco BRL) and re-suspended with PBS to a final concentration of 2 × 10^7^ cells/ml. Then, 100 μl cell suspension was inoculated subcutaneously in the right flank of 6-week-old female NOD/SCID mice (Vital River Laboratories, Beijing, China). When tumor volume reached approximately 150–250 mm^3^, mice bearing MKN45 cells were randomly assigned to treatment groups (*n* = 5) and given daily PBS (100 μl, by gavage), either AZD1775 (30 mg/kg/d, by gavage) alone or in combination with olaparib (25 mg/kg/d, ip) for 21 days. Tumor size and body weight were measured every 3 days and tumor volume (V) was calculated by formula: V = L × W^2^/2 (L, long diameter of the tumor; W, short diameter of the tumor). After the final drug administration, mice were sacrificed and tumors were stripped for successive assays. All animal experiments were approved by Peking University Cancer Hospital’s Institutional Animal Care and Use Committee and complied with the internationally-recognized Animal Research: Reporting of in vivo Experiments guideline.

### Immunohistochemistry (IHC)

After dewaxing, hydration, endogenous peroxidase removal and blocking with 5% BSA according to standard procedures, 4-μm thick formalin-fixed and paraffin-embedded (FFPE) sections were incubated with the primary anti-Ki-67 antibody (1:300) overnight at 4 °C followed by IgG/HRP polymer (ZSGB-BIO) and diaminobenzidine substrate (Gene Tech, Shanghai, China) complying to protocols. Staining results were independently evaluated by two pathologists from the department of pathology in Peking University Cancer Hospital & Institute as described in our previous study [24].

### Statistical analysis

All data were representative of 3 independent experiments and illustrated as means ± SD. Differences between groups were analyzed by one-way or repeated measures ANOVA using SPSS Version 20.0 software (SPSS Inc., Chicago, IL) (*P* < 0.05 was considered statistically significant).

## Results

### PARP inhibitor olaparib combined with WEE1/PLK1 dual inhibitor AZD1775 further reduces growth in GC cells

**Fig. 1:**
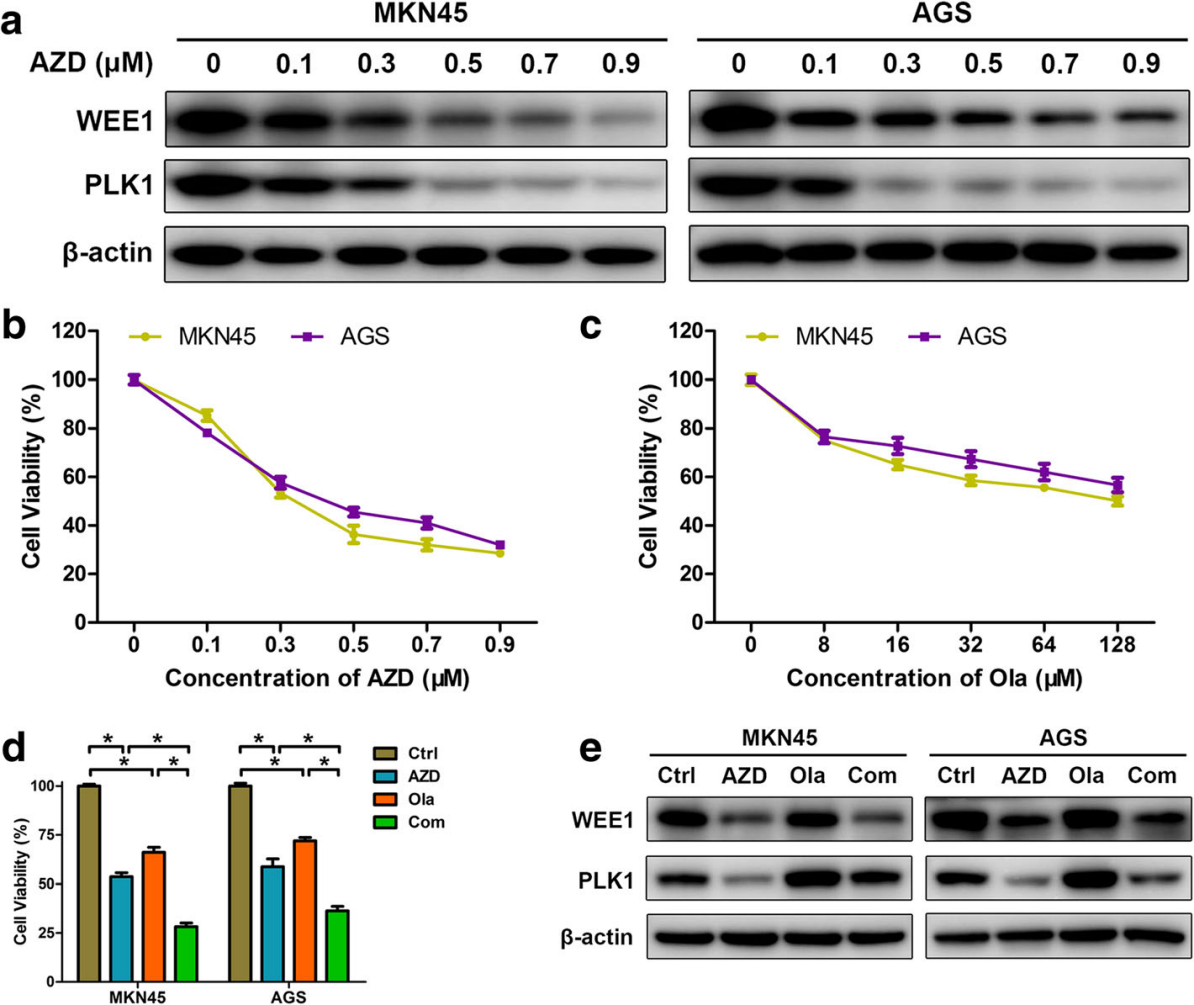
PARP inhibitor olaparib combined with WEE1/PLK1 dual inhibitor AZD1775 further reduces growth in GC cells. **a-c** MKN45 and AGS cells were treated with either olaparib or AZD1775 alone for 48 h in a dose-dependent manner. Protein extracts were probed with antibodies against WEE1 and PLK1 and CCK-8 assays were performed. **d** and **e** After exposure to 0.3 μM AZD1775 with/without 20 μM olaparib for 48 h, CCK-8 assays and immunoblots were performed in MKN45 and AGS cells. AZD, AZD1775; Ola, olaparib; Com, Combination. Data expressed as Mean ± SD and representative of three independent experiments. *, *P* < 0.05 by ANOVA analysis

As a widely-used WEE1 inhibitor, AZD1775 has recently proved functional for WEE1/PLK1 dual blockade [20]. Indeed, AZD1775 lowered WEE1 and PLK1 expressions in a dose-dependent manner in our GC cells (Fig. 1a). To evaluate therapeutic potentials of combined inhibition of PARP and WEE1/PLK1, GC cells were treated with either olaparib/AZD1775 alone or in combination. Monotherapy of either olaparib or AZD1775 reduced GC cell growth (Fig. 1b and c), while the combination of olaparib and AZD1775 intriguingly exerted higher growth inhibition than single-agent groups (Fig. 1d), indicating an augmented antitumor effect of PARP inhibitor in the presence of WEE1/PLK1 dual blockade against GC cells. Besides, the synergism between these two drugs was indicated by CI values less than 1 using the Chou-Talalay method (Additional file 1: Figure S1) [22]. Consistent with unchanged WEE1 levels in olaparib-treated GC cells, AZD1775-olaparib combination reduced WEE1 expressions to the same extent as AZD1775 monotherapy did compared to controls (Fig. 1e). In contrast, olaparib-induced PLK1 upregulations were attenuated by combined AZD1775 while PLK1 levels were higher in combination groups than AZD1775 alone. In line with previous observations that blockade of olaparib-upregulated PLK1 improved olaparib’s anticancer efficacy [21], potentiated cytotoxicity by olaparib plus AZD1775 might be, at least partially, due to PLK1 suppression by AZD1775 in GC cells.

### AZD1775 facilitates olaparib-induced apoptosis in GC cells

**Fig. 2:**
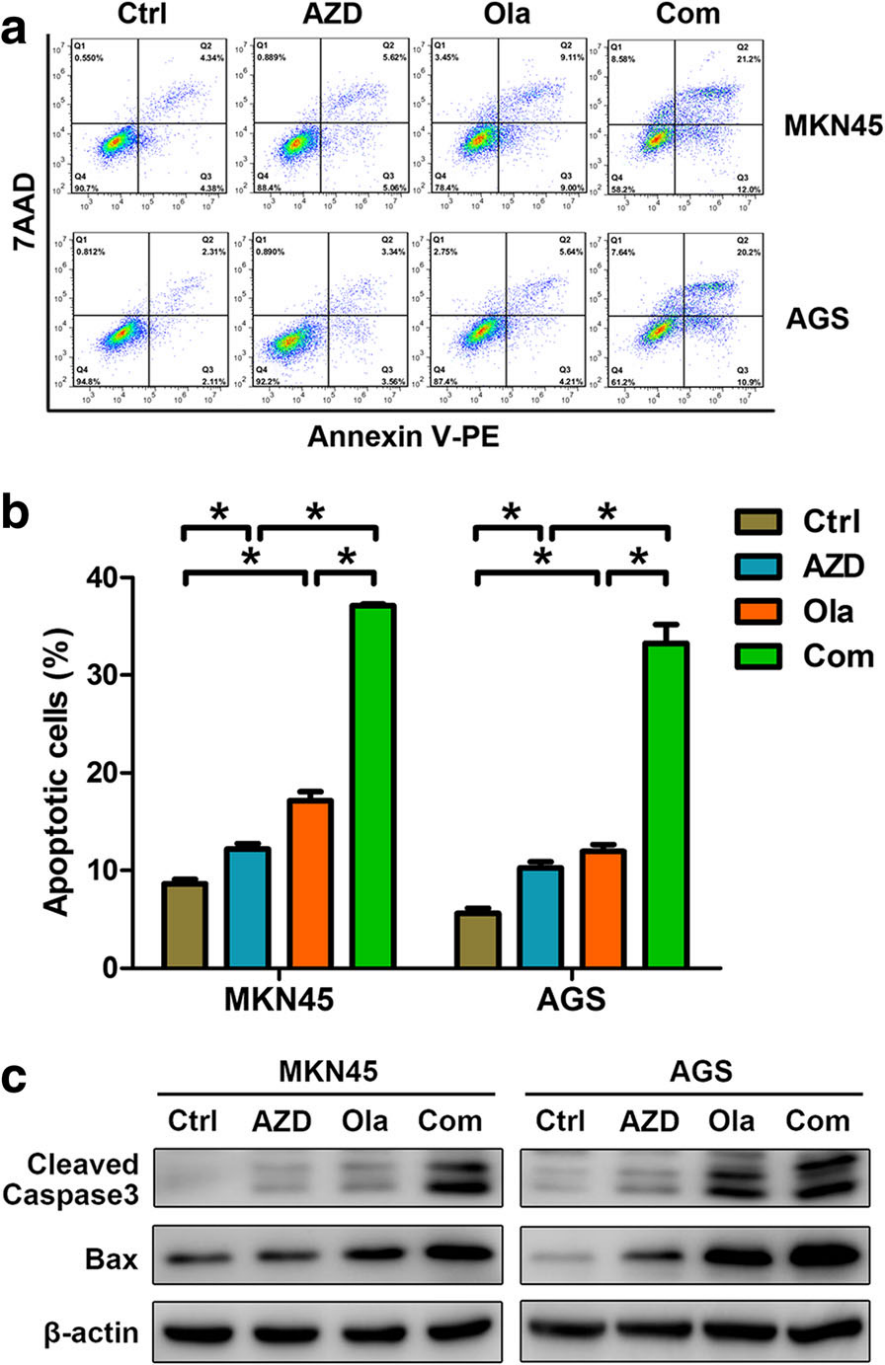
AZD1775 facilitates olaparib-induced apoptosis in GC cells. **a-c** Apoptosis analysis using Annexin V-PE/7-AAD double-staining and immunoblots with anti-caspase 3 and Bax antibodies were carried out in MKN45 and AGS cells treated with 0.3 μM AZD1775 in the presence or absence of 20 μM olaparib for 48 h. AZD, AZD1775; Ola, olaparib; Com, Combination. Data expressed as Mean ± SD and representative of three independent experiments. *, *P* < 0.05 by ANOVA analysis

Both PARP inhibitors and WEE1 inhibitors exert antitumor activity by inducing apoptosis [25, 26], so we assessed impacts of olaparib combined with AZD1775 on apoptosis. Compared to controls, AZD1775 or olaparib alone induced apoptosis to some extent, while more apoptosis was induced by their combination (Fig. 2a and b). Similarly, the cleavage of caspase3 and the expression of Bax were increased in GC cells exposed to AZD1775 or olaparib alone while further enhanced in combination groups (Fig. 2c). These data suggest that PARP inhibitor olaparib combined with WEE1/PLK1 dual inhibitor AZD1775 exerted strengthened pro-apoptotic capacity than their monotherapy in GC cells.

### WEE1/PLK1 blockade induces HR deficiency in GC cells

**Fig. 3:**
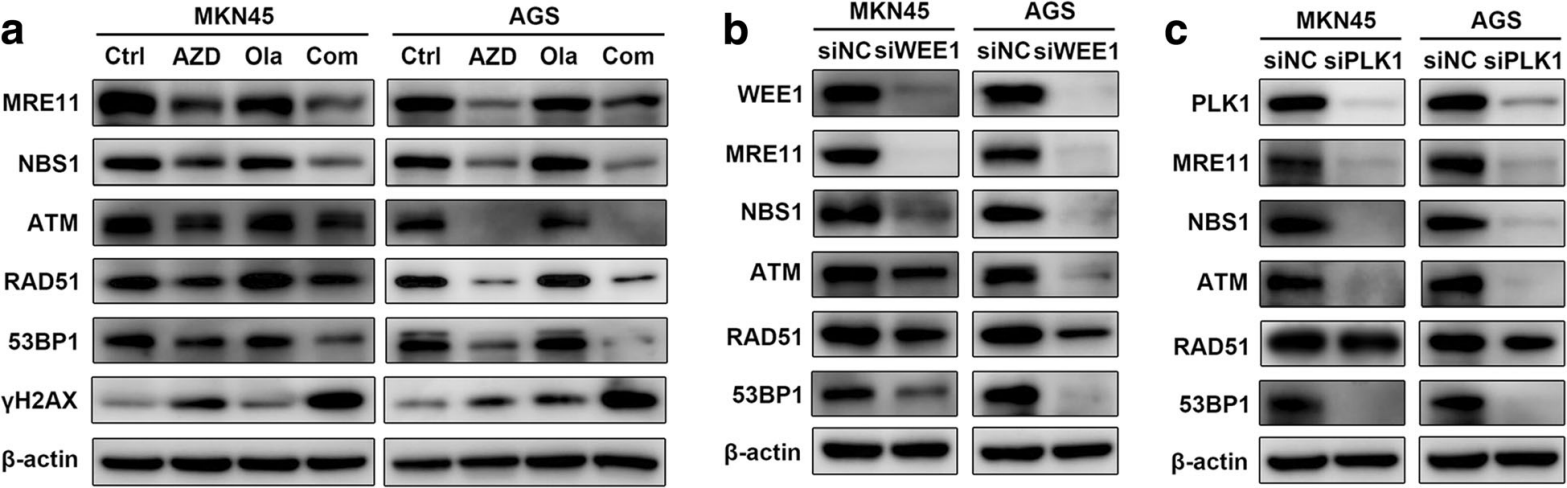
WEE1/PLK1 blockade induces HR deficiency in GC cells. **a** MKN45 and AGS cells were subjected to 0.3 μM AZD1775 with/without 20 μM olaparib for 48 h and protein extracts were probed with indicated antibodies for immunoblots. **b** and **c** Upon treatment of WEE1/PLK1 knockdown by siRNA, immunoblots were performed for WEE1, PLK1 and indicated HR-related proteins. AZD, AZD1775; Ola, olaparib; Com, Combination

WEE1 inhibition has been reported to cause defects in HR-mediated DDR [16–19], a potential predictive biomarker of PARP inhibitor sensitivity [6], suggesting that the enhanced cytotoxicity of olaparib combined with AZD1775 observed might be, at least partially, due to AZD1775’s effects on HR. Thus, changes of several key DDR factors involved in HR (MRE11, NBS1, ATM, RAD51 and 53BP1) were assessed. As expected, these HR-related molecules were downregulated by AZD1775 and largely unaffected by olaparib, while the effect and subsequent DNA damage marked by upregulated γH2AX were observed in AZD1775-olaparib combination compared to olaparib alone (Fig. 3a). These findings suggest that the HR impairment and DNA damage in response to AZD1775 administration remained in the presence of olaparib, enabling olaparib to be functional even in HR-proficient GC. When performing WEE1/PLK1 RNA knockdown for validation, the majority of HR-related proteins decreased after WEE1/PLK1 interference as in AZD1775 groups, emphasizing AZD1775-induced HR inhibition were dependent on WEE1/PLK1 repression (Fig. 3b and c). Taken together, AZD1775 disrupted HR and increased DNA damage via inducing WEE1/PLK1 dual inhibition, thus subsequently enhanced GC cells’ response to PARP inhibitors.

### AZD1775 abrogates olaparib-activated DNA damage checkpoint and causes mitotic DNA damage in GC cells

**Fig. 4:**
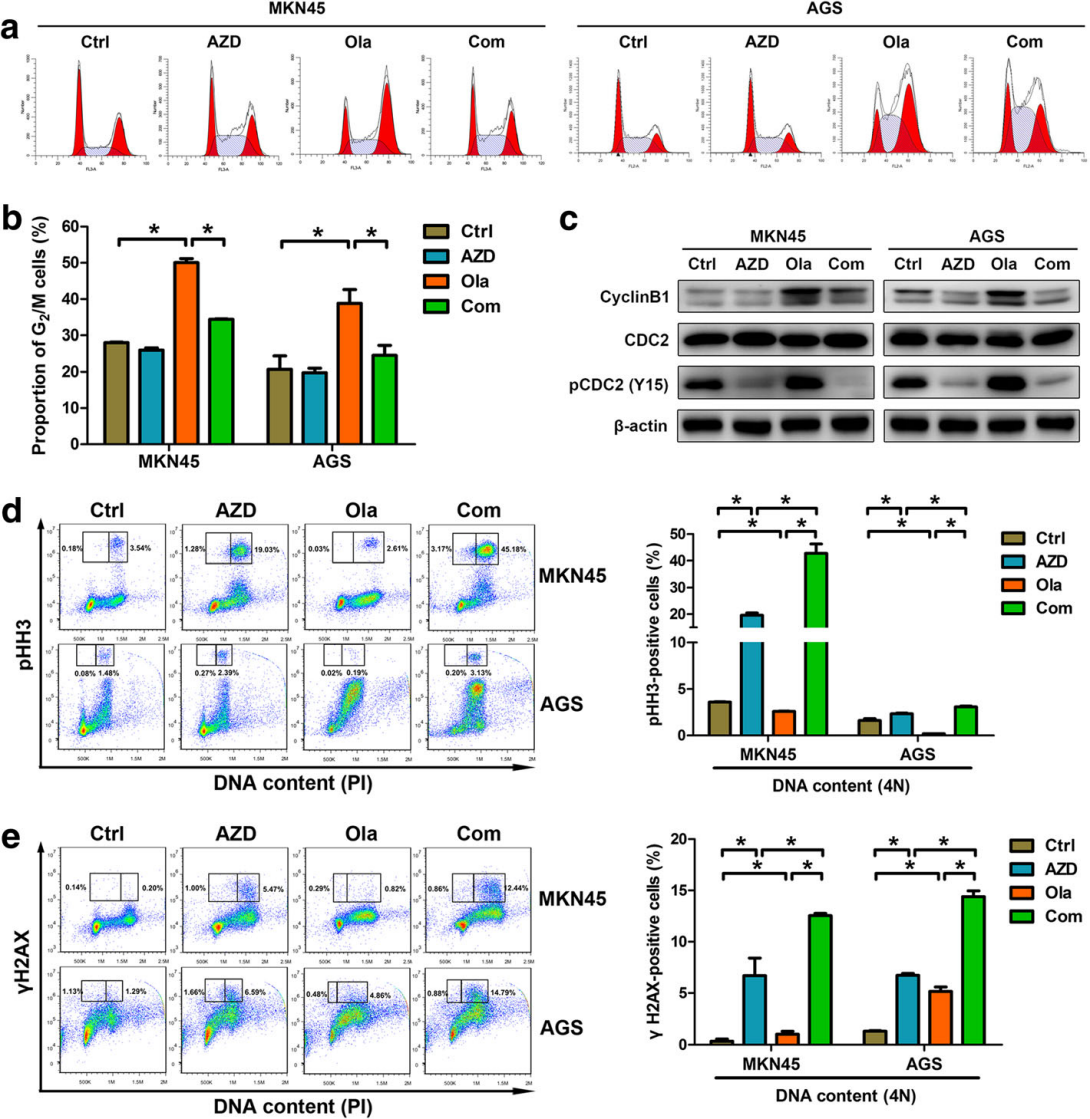
AZD1775 abrogates olaparib-activated DNA damage checkpoint and caused mitotic DNA damage in GC cells. Following cell cycle synchronization with serum deprivation for 24 h, 0.3 μM AZD1775 with/without 20 μM olaparib were exposed to MKN45 and AGS cells for 24 h. **a** and **b** Cell cycle was analyzed by flow cytometry. **c** Protein extracts were probed with anti-cyclin B1, CDC2 and pCDC2 antibodies. **d** and **e** pHH3 and γH2AX-positive cells at separate cell cycle stages were assessed by double-staining with pHH3/PI and γH2AX/PI, respectively, and quantified using FlowJo and ModFit software. AZD, AZD1775; Ola, olaparib; Com, Combination. Data expressed as Mean ± SD and representative of three independent experiments. *, *P* < 0.05 by ANOVA analysis

The DNA damage checkpoint, a pivotal target of WEE1 by CDC2 phosphorylation, stalls the cell cycle and protects cancer cells from death in response to unrepaired DNA lesions [14, 27]. Of note, PARP inhibition has been reported to activate the DNA damage checkpoint, which gave a rationale for synergy of PARP blockade with modulation that impaired DNA damage checkpoint [28]. Thus, we assessed a role of the DNA damage checkpoint disruption in AZD1775’s facilitation to olaparib’s antitumor activity. Our data show that olaparib did activate the DNA damage checkpoint marked by increased G_2_/M phase proportions and elevated cyclinB1/phosphorylated-CDC2 expressions, while AZD1775 intriguingly antagonized these effects (Fig. 4a-c). On the other hand, abrogation of DNA damage checkpoint is generally followed by mitotic DNA damage, particularly marked by the mitotic-related pHH3 and the DSB-related γH2AX changes [19, 29, 30]. Our data reveal that pHH3 or γH2AX staining in GC cells were significantly strengthened by AZD1775 while further enhanced by AZD1775-olaparib combination (Fig. 4d and e), validating the presence of an enhanced mitotic DNA damage in combination of AZD1775 and olaparib. Thus, administration of AZD1775 rescued the DNA damage checkpoint triggered by olaparib, which exerted stronger antigrowth efficacy than olaparib alone.

### AZD1775 enhances olaparib’s antitumor efficacy via HR defect in vivo

**Fig. 5:**
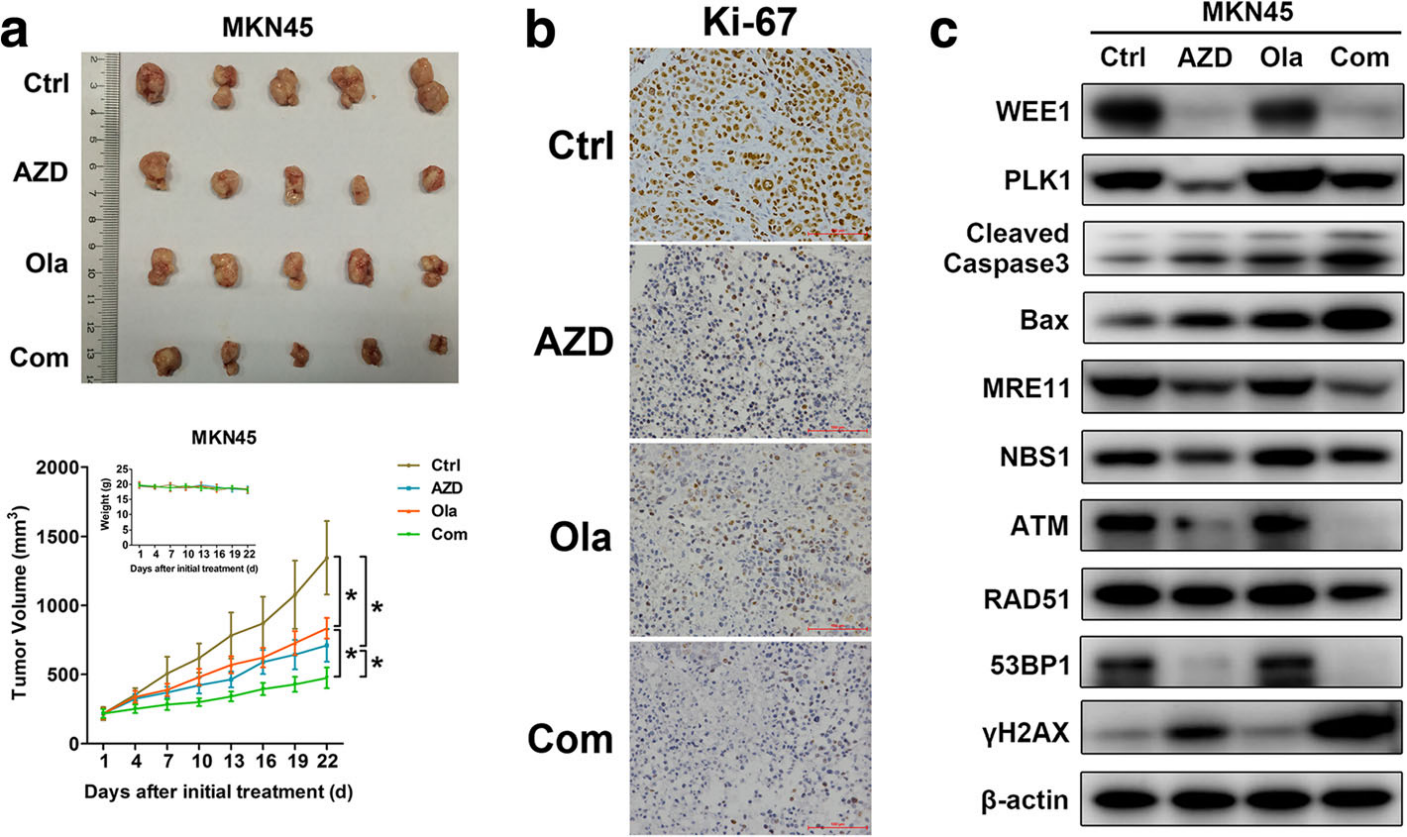
AZD1775 enhances olaparib’s antitumor efficacy via HR defect in vivo. AZD1775 (30 mg/kg/d, by gavage) with/without olaparib (25 mg/kg/d, ip) were given to mice bearing MKN45 tumors for 21 days (*n* = 5 per group). **a** Tumor volume was measured every 3 days after treatment and xenograft growth curves were shown. Data expressed as Mean ± SD. *P < 0.05 by repeated measures ANOVA analysis. **b** and **c** FFPE sections were stained with Ki-67 using IHC and tumor lysates were immunoblotted for indicated proteins. Original magnification, 200×. AZD, AZD1775; Ola, olaparib; Com, Combination

Considering the effectiveness of AZD1775-olaparib combination scheme observed in vitro, we assessed whether these effects extended to in vivo contexts. Compared to the control group, AZD1775-olaparib combination achieved higher inhibition in gastric tumor growth than their monotherapy (Fig. 5a). The lowest overall proliferation rate (Ki-67 immunostaining, Fig. 5b) and the highest pro-apoptotic capacity (cleaved caspase3 and Bax, Fig. 5c) were also observed in the combination group, further validating the improved anticancer efficiency of AZD1775 plus olaparib in mice bearing GC tumors. On molecular levels, the WEE1/PLK1 dual inhibitor AZD1775 induced HR deficiency (marked by reduced MRE11, NBS1, ATM, 53BP1 and RAD51), leading to DNA damage (marked by γH2AX) in GC xenografts as seen in vitro (Fig. 5c). Notably, these antitumor actions were stronger in the combined group compared to olaparib alone (Fig. 5c). In conclusion, our study revealed that AZD1775 augmented olaparib’s therapeutic efficiency in GC xenografts, at least partially through disrupting HR-mediated DDR.

## Discussion

Despite that targeted therapy has unveiled a new era to cancer treatment, current strategies for advanced GC is insufficient to remarkably improve patients’ prognosis. Hence, identifying potential novel targets and developing new combination strategies are critical to benefit new subgroups and optimize effectiveness of current therapy against GC. PARP inhibitors targeting DDR pathways exhibit potent anticancer activity in preclinical models and clinical studies of GC, especially in those with low ATM or RAD51 expression [4, 31, 32]. Due to a close association between efficacy of PARP inhibitors and HR impairment, more and more combination strategies with HR deficiency inducers have been developed against many cancers [8–13]. However, dual inhibition of PARP and HR functions has not yet been investigated in GC.

Of interest, targeting WEE1, an alternative promising strategy against GC [15], can impair HR functions and is proposed to be a sensitizer of PARP inhibitors [14]. For the first time, our data show that by inducing growth inhibition and apoptosis, combination of the PARP inhibitor olaparib with the WEE1/PLK1 dual inhibitor AZD1775 exerted evidently enhanced antitumor effects in GC cell lines and xenografts than either olaparib or AZD1775 alone (Figs. 1, 2 and 5). Apart from efficacy, safety and tolerability are also key issues in the treatment of GC, especially for combination strategies. Olaparib has been approved to treat BRCA-mutated ovarian cancer at the dose of 400 mg twice a day [33] and proved well tolerated for patients with advanced GC at the dose of 100 mg twice daily in clinical trials [4, 34]. Given the clinical data and olaparib’s daily dose of 25-100 mg/kg extensively-used in mice experiments [13, 32, 35–37], 25 mg/kg equal to about 140-150 mg in human with 70 kg of body weight [38, 39] was applied in our GC mice studies. As for AZD1775, the human maximum tolerated dose of 225 mg twice daily [40, 41] equates to a rough 79 mg/kg daily dose for mice. Based on previous mice experiments [42–46], daily oral administration of 30 mg/kg AZD1775 for 3 weeks were used in our work. Consistently, our GC xenograft models had no weight loss after exposure to olaparib (25 mg/kg/d) with/without AZD1775 (30 mg/kg/d) (Fig. 5a), lower than their clinically achievable levels, suggesting safety and tolerability of this combination strategy in GC.

**Fig. 6:**
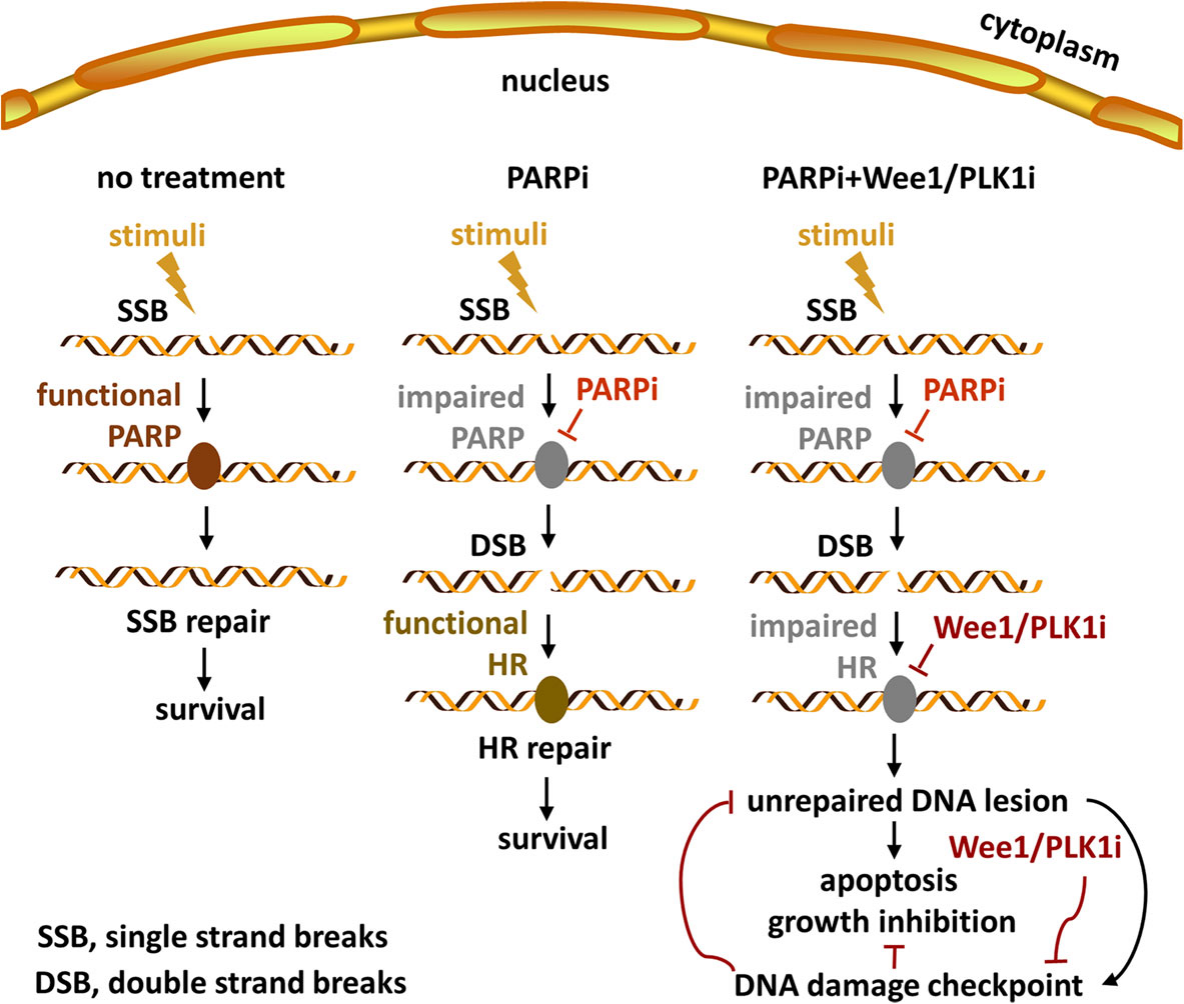
Representation of potentiated anticancer activity by PARP inhibitor combined with WEE1/PLK1 dual inhibitor against GC. PARP inhibitors prevent repairs of SSB that convert into DSB in the context of functional HR, which limits efficacy of PARP inhibitors against GC. In the presence of WEE1/PLK1 dual inhibitors, HR can be impaired and DNA damage checkpoint be inactivated to promote lethality of GC cells subjected to PARP inhibitors

WEE1 repression has been reported to compromise HR functions marked by reduced RAD51 and 53BP1 along with subsequent accumulated γH2AX [16–19]. Consistently, our findings manifest a downregulation of RAD51 and 53BP1 and an upregulation of γH2AX in GC cell lines and xenografts treated with AZD1775 (Fig. 3a and 5c). RAD51 and 53BP1 was also decreased by olaparib in the presence of AZD1775 compared to olaparib alone, further confirming that AZD1775 potentiated olaparib’s efficiency in GC via impairing HR. Alternative HR-related proteins, such as MRE11, NBS1 and ATM, have been also reportedly lowered by modulation that disrupts HR to improve antitumor efficacy of PARP inhibitors [11, 12]. Resembling RAD51 and 53BP1, responses of these factors to olaparib were replenished by AZD1775 administration (Fig. 3a and 5c), emphasizing that HR impairment was indispensable for the augmented anticancer activity of AZD1775-olaparib combination. As reported, PARP inhibitors primarily blocked repairs of SSB to exert anticancer activity; however, SSB converted into DSB, which could be repaired by HR feedback reactions to impede PARP inhibitors’ responses in HR-functional populations [2, 3]. As an improvement, our data show that AZD1775 in combined therapy caused HR impairment and prevented DSB from repairs to augment DNA damage-mediated cytotoxicity against GC. WEE1/PLK1 blockade provided a HR-defective context, which served as a replenishment to PARP inhibitor functions and gave a rationale for the enhanced antitumor efficacy of the AZD1775-olaparib combination scheme (Fig. 6). Of note, HR dysfunctions that provide an opportunity of optimizing PARP inhibitors’ antitumor efficiency are prevalently evaluated by defective total HR-mediated DDR protein levels beyond which phosphorylation status or immunofluorescent foci of these proteins as well as HR function assays are widely-used methods to monitor HR activity [12, 13, 17, 25, 47]. Thus, other HR function experiments warrant HR impairment’s contribution to enhanced efficacy of AZD1775 plus olaparib against GC in future studies. Besides, PARP trapping serves as key actions for PARP inhibitors in the treatment of cancer [2, 23] and thus we also assessed effects of AZD1775-olaparib combination on it using methods as previously described [23]. Additional file 2: Figure S2b demonstrates olaparib-mediated trapping of PARP to chromatin marked by an increased ratio of trapped PARP1 to total PARP1, however, AZD1775 had no PARP trapping ability and failed to augment olaparib-induced PARP trapping in GC cells. Similarly, PARP catalytic activity, another pivotal factor of PARP inhibitors’ cytotoxicity [23], was inhibited by olaparib indicated by a robust reduction of PARs compared to controls whereas AZD1775 almost had no impact in GC cells (Additional file 2: Figure S2b). Taken together, PARP trapping to chromatin and PARP catalytic inhibition were responsible for PARP inhibitors’ efficacy but dispensable for augmented cytotoxicity of olaparib-AZD1775 combination in GC. Since SSB accumulations followed by replication stress enhancement are a major cause behind single-agent antitumor activity of AZD1775 [23, 46], we also investigated AZD1775-olaparib combination’s impacts on replication stress-associated ATR/Chk1 cascade. Additional file 2: Figure S2a revealed AZD1775-enhanced ATR/PRA32/Chk1 phosphorylation and similar/a bit larger effects when combined with olaparib (phosphorylation sites of PRA32 or Chk1 used are ATR/WEE1 dependent [23, 48–50]). Thus, AZD1775 induced excessive SSB accumulations and replication stress in our GC cells consistent with previous publications [23, 49, 51], which may facilitate SSB’s conversion to DSB and ensuing AZD1775-induced HR defect in synthetic lethality with olaparib.

Lethality of disrupting DNA damage repairs can be mitigated by functional DNA damage checkpoint (a.k.a. G_2_/M checkpoint) that allows DNA repairs before mitotic entry and keeps cancer cells with unrepaired DNA lesions from death [14, 27]. WEE1 inhibitors primarily target DNA damage checkpoint [14, 27], enabling DNA damage checkpoint inactivation to augment lethality of HR deficiency in the context of dual PARP/WEE1 inhibition. As marked by an alteration in G_2_/M checkpoint-related proteins (upregulated cyclinB1 and CDC2 phosphorylation; Fig. 4a-c), olaparib-induced G_2_/M arrest resulted from an activation of G_2_/M checkpoint [18, 28]. WEE1 inhibition has been reported to improve efficacy of G_2_ arrest inducers through G_2_/M checkpoint inactivation [18, 52], which is dependent on WEE1 blockade’s effects on CDC2 de-phosphorylation [18, 29, 53]. In our GC cells, AZD1775 abrogated olaparib-activated G_2_/M checkpoint through CDC2 de-phosphorylation and AZD1775-olaparib combination forced mitotic progression with unrepaired DNA lesions (Fig. 4c-e), called mitotic DNA damage that is responsible for an potentiated anticancer efficiency of AZD1775-combined strategies [18, 19, 29, 52–54]. Beyond mitotic DNA damage, well-described AZD1775-mediated prolonged mitosis also contributes to pHH3 increment and apoptosis seen in our work (Fig. 2 and 4d). As anticipated, mitosis exit after nocodazole release (marked by a gradual decline in cyclinB1) [55, 56] could be impeded by AZD1775 in our models, which relied on AZD1775-mediated CDC2 de-phosphorylation/activation (Additional file 2: Figure S2c). These data suggest presence of AZD1775-mediated prolonged mitosis in GC cells [55, 57]. In the context of forced mitotic progression and prolonged mitosis induced by AZD1775, AZD1775-olaparib combination ensued to accumulate DNA damage and increase apoptosis (Figs. 2, 5 and 6). Consistent with our data, DNA damage-associated apoptosis can be mediated by HR defect and DNA damage checkpoint inactivation [10–12, 14, 26, 30]. Taken together, olaparib plus AZD1775 augmented DNA damage-mediated growth inhibition and apoptotic cell death by disrupting HR functions and the DNA damage checkpoint in GC.

Recently, dual targeting of PLK1 and WEE1 has been reported to elicit AZD1775’s single agent cytotoxicity in lung cancer [20]. However, molecular mechanisms underlying AZD1775-decresed exprexssion of the new target PLK1 as observed in our study (Fig. 1a, e and 5c) remain uninvestigated. Intriguingly, several AZD1775’s functions can be partially explained by PLK1 inhibition. For one thing, AZD1775-reduced PLK1 may contribute to AZD1775-induced DNA damage and apoptosis still presented in WEE1 CRISPR-Cas9 knockout cancer cells [20], in line with reliance of AZD1775’s cytotoxicity on targeting PLK1 and WEE1 in our GC cells (Figs. 1 and 2). We also for the first time demonstrated that PLK1 knockdown lowered HR-related proteins as WEE1 knockdown and AZD1775 (Fig. 3), suggesting that PLK1 inhibition-mediated HR defect potentiated DNA damage-associated anticancer efficacy of olaparib plus AZD1775 against GC, resembling WEE1 blockade. For another, the serine/threonine kinase PLK1, governs several mitotic steps, especially mitotic exit and entry into mitosis, keeping cancer cells from genome instability [58]. The spindle assembly checkpoint activation that often delays mitotic exit can be triggered by potent PLK1 inhibitors like AZD1775 and WEE1 knockdown [55, 57, 58], which gives a rationale for AZD1775-prolonged mitosis (Additional file 2: Figure S2c). Of note, PLK1 blockade can directly inhibit WEE1’s phosphorylation and ensuing proteasomal degradation [14] to yield opposite effects on CDC2 activity and DNA damage checkpoint to WEE1 knockdown and AZD1775 (Fig. 4). Moreover, suppression of olaparib-induced PLK1 upregulation can optimize olaparib’s anticancer efficacy in p53-mutated prostate cancer rather than those with p53 wild type [21]. AZD1775 plus olaparib had similar effects in our p53 wild type GC cells (MKN45 and AGS; Fig. 1) despite unknown mechanisms underlying the discrepancy. Thus, whether dual PLK1/WEE1 inhibition elicits anticancer efficacy superior to WEE1 blockade, especially in the presence of olaparib in GC, merits further investigations.

## Conclusions

By disrupting DDR pathways and the DNA damage checkpoint to increase DNA damage and apoptosis, WEE1/PLK1 dual inhibitors augmented PARP inhibitors’ antitumor activity in GC cells and xenografts. Hence, the combination scheme with WEE1/PLK1 dual inhibitors may potentially optimize PARP inhibitor’s effect against GC and extend PARP inhibitor’s accessibility from HR-defective to a larger range of HR-proficient patients, which will be helpful for future clinical settings on cancer targeted therapy.

## Additional files

Additional file 1: **Figure S1.** Synergistic growth-inhibition between AZD1775 and olaparib in GC cells. After GC cells were treated with combination of AZD1775 and olaparib at a fixed ratio of 1:200 (AZD1775: olaparib) for 48 h, cell viability were measured by CCK-8 assays and F_a_-CI plots were made using the Chou-Talalay method. CI < 1, =1 and > 1 indicated synergism, additivity, and antagonism, respectively. (TIF 206 kb)

Additional file 2: **Figure S2.** Effects of AZD1775 plus olaparib on SSB accumulations, PARP trapping and prolonged mitosis in GC cells. (a-c) after drug treatment as indicated, proteins extracted from whole cell lysates or chromatin were probed with indicated antibodies. Trapped/total PARP1 indicated the ratios of PARP1 levels in chromatin to PARP1 levels in whole cell lysates which were then normalized to controls. Noco, Nocodazole. (TIF 647 kb)

## Abbreviations

7-AAD: 7-amino-actinomycin
DDR: DNA damage repair
DSB: Double-strand DNA breaks
FFPE: Formalin-fixed and paraffin-embedded
GC: Gastric cancer
HR: Homologous recombination
IHC: Immunohistochemistry
PARP: Poly ADP-ribose polymerase
PE: Phycoerythrin
PI: Propidium iodide
PLK1: Polo-like kinase 1
SSB: Single-strand DNA breaks

## References

[1] Chen W, Zheng R, Baade PD, Zhang S, Zeng H, Bray F (2016). Cancer statistics in China, 2015. CA Cancer J Clin.

[2] Pommier Y, O'Connor MJ, de Bono J (2016). Laying a trap to kill cancer cells: PARP inhibitors and their mechanisms of action. Sci Transl Med.

[3] Shen J, Peng Y, Wei L, Zhang W, Yang L, Lan L (2015). ARID1A deficiency impairs the DNA damage checkpoint and sensitizes cells to PARP inhibitors. Cancer Discov.

[4] Bang YJ, Im SA, Lee KW, Cho JY, Song EK, Lee KH (2015). Randomized, double-blind phase II trial with prospective classification by ATM protein level to evaluate the efficacy and tolerability of Olaparib plus paclitaxel in patients with recurrent or metastatic gastric Cancer. J Clin Oncol.

[5] Lin KY, Kraus WL (2017). PARP inhibitors for Cancer therapy. Cell.

[6] Lord CJ, Ashworth A (2017). PARP inhibitors: synthetic lethality in the clinic. Sicence.

[7] Morra F, Luise C, Visconti R, Staibano S, Merolla F, Ilardi G (2015). New therapeutic perspectives in CCDC6 deficient lung cancer cells. Int J Cancer.

[8] Amin O, Beauchamp MC, Nader PA, Laskov I, Iqbal S, Philip CA (2015). Suppression of homologous recombination by insulin-like growth factor-1 inhibition sensitizes cancer cells to PARP inhibitors. BMC Cancer.

[9] Anderson VE, Walton MI, Eve PD, Boxall KJ, Antoni L, Caldwell JJ (2011). CCT241533 is a potent and selective inhibitor of CHK2 that potentiates the cytotoxicity of PARP inhibitors. Cancer Res.

[10] Johnson N, Li YC, Walton ZE, Cheng KA, Li D, Rodig SJ (2011). Compromised CDK1 activity sensitizes BRCA-proficient cancers to PARP inhibition. Nat Med.

[11] Rasmussen RD, Gajjar MK, Jensen KE, Hamerlik P (2016). Enhanced efficacy of combined HDAC and PARP targeting in glioblastoma. Mol Oncol.

[12] Sun C, Fang Y, Yin J, Chen J, Ju Z, Zhang D (2017). Rational combination therapy with PARP and MEK inhibitors capitalizes on therapeutic liabilities in RAS mutant cancers. Sci Transl Med.

[13] Yang L, Zhang Y, Shan W, Hu Z, Yuan J, Pi J (2017). Repression of BET activity sensitizes homologous recombination-proficient cancers to PARP inhibition. Sci Transl Med.

[14] Matheson CJ, Backos DS, Reigan P (2016). Targeting WEE1 kinase in Cancer. Trends Pharmacol Sci.

[15] Kim HY, Cho Y, Kang H, Yim YS, Kim SJ, Song J (2016). Targeting the WEE1 kinase as a molecular targeted therapy for gastric cancer. Oncotarget.

[16] Kausar T, Schreiber JS, Karnak D, Parsels LA, Parsels JD, Davis MA (2015). Sensitization of pancreatic cancers to gemcitabine Chemoradiation by WEE1 kinase inhibition depends on homologous recombination repair. Neoplasia.

[17] Krajewska M, Heijink AM, Bisselink YJ, Seinstra RI, Sillje HH, de Vries EG (2013). Forced activation of Cdk1 via wee1 inhibition impairs homologous recombination. Oncogene.

[18] Wang Z, Lai ST, Ma NY, Deng Y, Liu Y, Wei DP (2015). Radiosensitization of metformin in pancreatic cancer cells via abrogating the G2 checkpoint and inhibiting DNA damage repair. Cancer Lett.

[19] Karnak D, Engelke CG, Parsels LA, Kausar T, Wei D, Robertson JR (2014). Combined inhibition of Wee1 and PARP1/2 for radiosensitization in pancreatic cancer. Clin Cancer Res.

[20] Wright G, Golubeva V, Remsing Rix LL, Berndt N, Luo Y, Ward GA (2017). Dual targeting of WEE1 and PLK1 by AZD1775 elicits single agent cellular anticancer activity. ACS Chem Biol.

[21] Li J, Wang R, Kong Y, Broman MM, Carlock C, Chen L (2017). Targeting Plk1 to enhance efficacy of Olaparib in castration-resistant prostate Cancer. Mol Cancer Ther.

[22] Chou TC (2006). Theoretical basis, experimental design, and computerized simulation of synergism and antagonism in drug combination studies. Pharmacol Rev.

[23] Parsels LA, Karnak D, Parsels JD, Zhang Q, Velez-Padilla J, Reichert ZR (2018). PARP1 trapping and DNA replication stress enhance Radiosensitization with combined WEE1 and PARP inhibitors. Mol Cancer Res.

[24] Zhu Y, Tian T, Zou J, Wang Q, Li Z, Li Y (2015). Dual PI3K/mTOR inhibitor BEZ235 exerts extensive antitumor activity in HER2-positive gastric cancer. BMC Cancer.

[25] Xia Q, Cai Y, Peng R, Wu G, Shi Y, Jiang W (2014). The CDK1 inhibitor RO3306 improves the response of BRCA-pro fi cient breast cancer cells to PARP inhibition. Int J Oncol.

[26] Kreahling JM, Gemmer JY, Reed D, Letson D, Bui M, Altiok S (2012). MK1775, a selective Wee1 inhibitor, shows single-agent antitumor activity against sarcoma cells. Mol Cancer Ther.

[27] Do K, Doroshow JH, Kummar S (2013). Wee1 kinase as a target for cancer therapy. Cell Cycle.

[28] Yin Y, Shen Q, Zhang P, Tao R, Chang W, Li R (2017). Chk1 inhibition potentiates the therapeutic efficacy of PARP inhibitor BMN673 in gastric cancer. Am J Cancer Res.

[29] Osman AA, Monroe MM, Ortega Alves MV, Patel AA, Katsonis P, Fitzgerald AL (2015). Wee-1 kinase inhibition overcomes cisplatin resistance associated with high-risk TP53 mutations in head and neck cancer through mitotic arrest followed by senescence. Mol Cancer Ther.

[30] Lal S, Zarei M, Chand SN, Dylgjeri E, Mambelli-Lisboa NC, Pishvaian MJ (2016). WEE1 inhibition in pancreatic cancer cells is dependent on DNA repair status in a context dependent manner. Sci Rep.

[31] Kubota E, Williamson CT, Ye R, Elegbede A, Peterson L, Lees-Miller SP (2014). Low ATM protein expression and depletion of p53 correlates with olaparib sensitivity in gastric cancer cell lines. Cell Cycle.

[32] Min A, Im SA, Yoon YK, Song SH, Nam HJ, Hur HS (2013). RAD51C-deficient cancer cells are highly sensitive to the PARP inhibitor olaparib. Mol Cancer Ther.

[33] Deeks ED (2015). Olaparib: first global approval. Drugs.

[34] Bang YJ, Xu RH, Chin K, Lee KW, Park SH, Rha SY (2017). Olaparib in combination with paclitaxel in patients with advanced gastric cancer who have progressed following first-line therapy (GOLD): a double-blind, randomised, placebo-controlled, phase 3 trial. Lancet Oncol.

[35] Bian X, Gao J, Luo F, Rui C, Zheng T, Wang D (2018). PTEN deficiency sensitizes endometrioid endometrial cancer to compound PARP-PI3K inhibition but not PARP inhibition as monotherapy. Oncogene.

[36] Pignochino Y, Capozzi F, D'Ambrosio L, Dell'Aglio C, Basirico M, Canta M (2017). PARP1 expression drives the synergistic antitumor activity of trabectedin and PARP1 inhibitors in sarcoma preclinical models. Mol Cancer.

[37] Schoonen PM, Talens F, Stok C, Gogola E, Heijink AM, Bouwman P (2017). Progression through mitosis promotes PARP inhibitor-induced cytotoxicity in homologous recombination-deficient cancer cells. Nat Commun.

[38] Kundu P, Genander M, Straat K, Classon J, Ridgway RA, Tan EH (2015). An EphB-Abl signaling pathway is associated with intestinal tumor initiation and growth. Sci Transl Med.

[39] Reagan-Shaw S, Nihal M, Ahmad N (2008). Dose translation from animal to human studies revisited. FASEB J.

[40] Do K, Wilsker D, Ji J, Zlott J, Freshwater T, Kinders RJ (2015). Phase I study of single-agent AZD1775 (MK-1775), a Wee1 kinase inhibitor, in patients with refractory solid tumors. J Clin Oncol.

[41] Leijen S, van Geel RM, Pavlick AC, Tibes R, Rosen L, Razak AR (2016). Phase I study evaluating WEE1 inhibitor AZD1775 as monotherapy and in combination with gemcitabine, cisplatin, or carboplatin in patients with advanced solid tumors. J Clin Oncol.

[42] Rajeshkumar NV, De Oliveira E, Ottenhof N, Watters J, Brooks D, Demuth T (2011). MK-1775, a potent Wee1 inhibitor, synergizes with gemcitabine to achieve tumor regressions, selectively in p53-deficient pancreatic cancer xenografts. Clin Cancer Res.

[43] Chang Q, Chandrashekhar M, Ketela T, Fedyshyn Y, Moffat J, Hedley D (2016). Cytokinetic effects of Wee1 disruption in pancreatic cancer. Cell Cycle.

[44] Russell MR, Levin K, Rader J, Belcastro L, Li Y, Martinez D (2013). Combination therapy targeting the Chk1 and Wee1 kinases shows therapeutic efficacy in neuroblastoma. Cancer Res.

[45] Van Linden AA, Baturin D, Ford JB, Fosmire SP, Gardner L, Korch C (2013). Inhibition of Wee1 sensitizes cancer cells to antimetabolite chemotherapeutics in vitro and in vivo, independent of p53 functionality. Mol Cancer Ther.

[46] Guertin AD, Li J, Liu Y, Hurd MS, Schuller AG, Long B (2013). Preclinical evaluation of the WEE1 inhibitor MK-1775 as single-agent anticancer therapy. Mol Cancer Ther.

[47] Krumm A, Barckhausen C, Kucuk P, Tomaszowski KH, Loquai C, Fahrer J (2016). Enhanced histone deacetylase activity in malignant melanoma provokes RAD51 and FANCD2-triggered drug resistance. Cancer Res.

[48] Liu S, Opiyo SO, Manthey K, Glanzer JG, Ashley AK, Amerin C (2012). Distinct roles for DNA-PK, ATM and ATR in RPA phosphorylation and checkpoint activation in response to replication stress. Nucleic Acids Res.

[49] Jin J, Fang H, Yang F, Ji W, Guan N, Sun Z (2018). Combined inhibition of ATR and WEE1 as a novel therapeutic strategy in triple-negative breast Cancer. Neoplasia.

[50] Massey AJ (2016). Inhibition of ATR-dependent feedback activation of Chk1 sensitises cancer cells to Chk1 inhibitor monotherapy. Cancer Lett.

[51] Saini P, Li Y, Dobbelstein M (2015). Wee1 is required to sustain ATR/Chk1 signaling upon replicative stress. Oncotarget.

[52] Sarcar B, Kahali S, Prabhu AH, Shumway SD, Xu Y, Demuth T (2011). Targeting radiation-induced G(2) checkpoint activation with the Wee-1 inhibitor MK-1775 in glioblastoma cell lines. Mol Cancer Ther.

[53] Wang G, Niu X, Zhang W, Caldwell JT, Edwards H, Chen W (2015). Synergistic antitumor interactions between MK-1775 and panobinostat in preclinical models of pancreatic cancer. Cancer Lett.

[54] Bridges KA, Hirai H, Buser CA, Brooks C, Liu H, Buchholz TA (2011). MK-1775, a novel Wee1 kinase inhibitor, radiosensitizes p53-defective human tumor cells. Clin Cancer Res.

[55] Visconti R, Della Monica R, Palazzo L, D'Alessio F, Raia M, Improta S (2015). The Fcp1-Wee1-Cdk1 axis affects spindle assembly checkpoint robustness and sensitivity to antimicrotubule cancer drugs. Cell Death Differ.

[56] Chang WL, Yu CC, Chen CS, Guh JH (2015). Tubulin-binding agents down-regulate matrix metalloproteinase-2 and -9 in human hormone-refractory prostate cancer cells - a critical role of Cdk1 in mitotic entry. Biochem Pharmacol.

[57] Lewis CW, Jin Z, Macdonald D, Wei W, Qian XJ, Choi WS (2017). Prolonged mitotic arrest induced by Wee1 inhibition sensitizes breast cancer cells to paclitaxel. Oncotarget.

[58] Choi M, Kim W, Cheon MG, Lee CW, Kim JE (2015). Polo-like kinase 1 inhibitor BI2536 causes mitotic catastrophe following activation of the spindle assembly checkpoint in non-small cell lung cancer cells. Cancer Lett.

