# Supplementary figures and images for: Augmented antitumor activity by olaparib plus AZD1775 in gastric cancer through disrupting DNA damage repair pathways and DNA damage checkpoint
Source: J Exp Clin Cancer Res. 2018 Jun 28;37:129. doi: 10.1186/s13046-018-0790-7 (PMC6027790; doi:10.1186/s13046-018-0790-7)

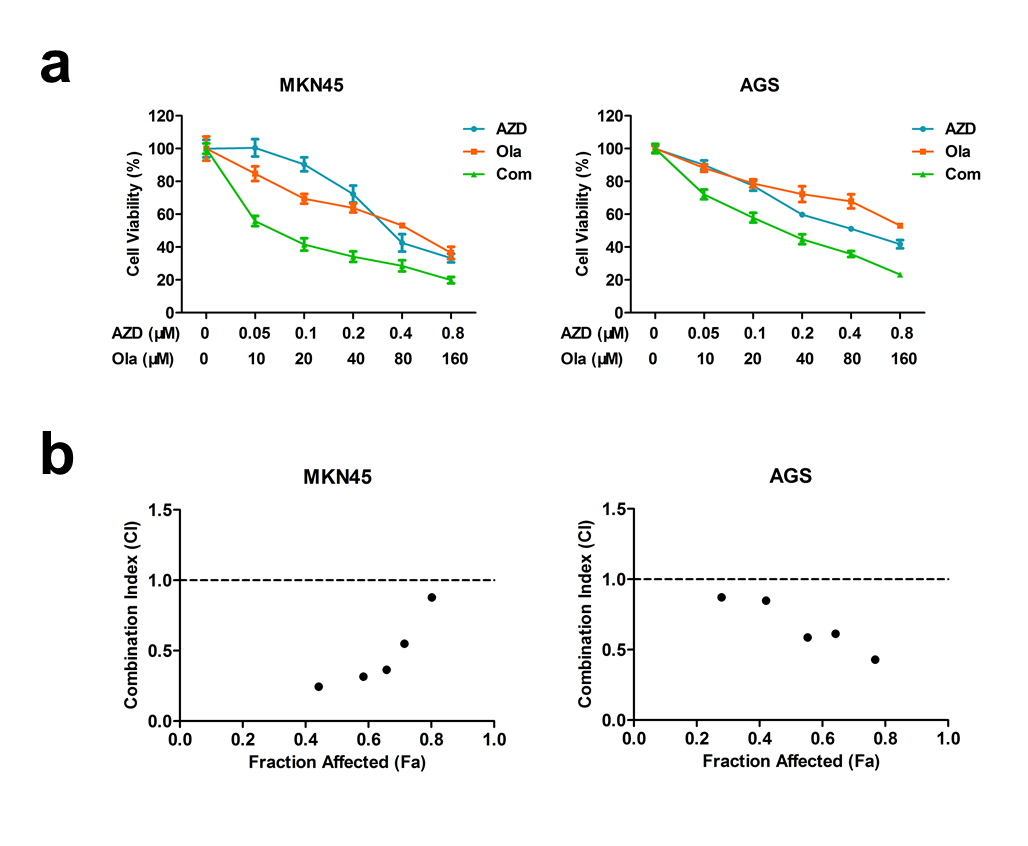

Supplement: Supplementary file 1 — Figure S1. Synergistic growth-inhibition between AZD1775 and olaparib in GC cells. After GC cells were treated with combination of AZD1775 and olaparib at a fixed ratio of 1:200 (AZD1775: olaparib) for 48 h, cell viability were measured by CCK-8 assays and Fa-CI plots were made using the Chou-Talalay method. CI < 1, =1 and > 1 indicated synergism, additivity, and antagonism, respectively. (TIF 206 kb) [file 13046_2018_790_MOESM1_ESM.tif]

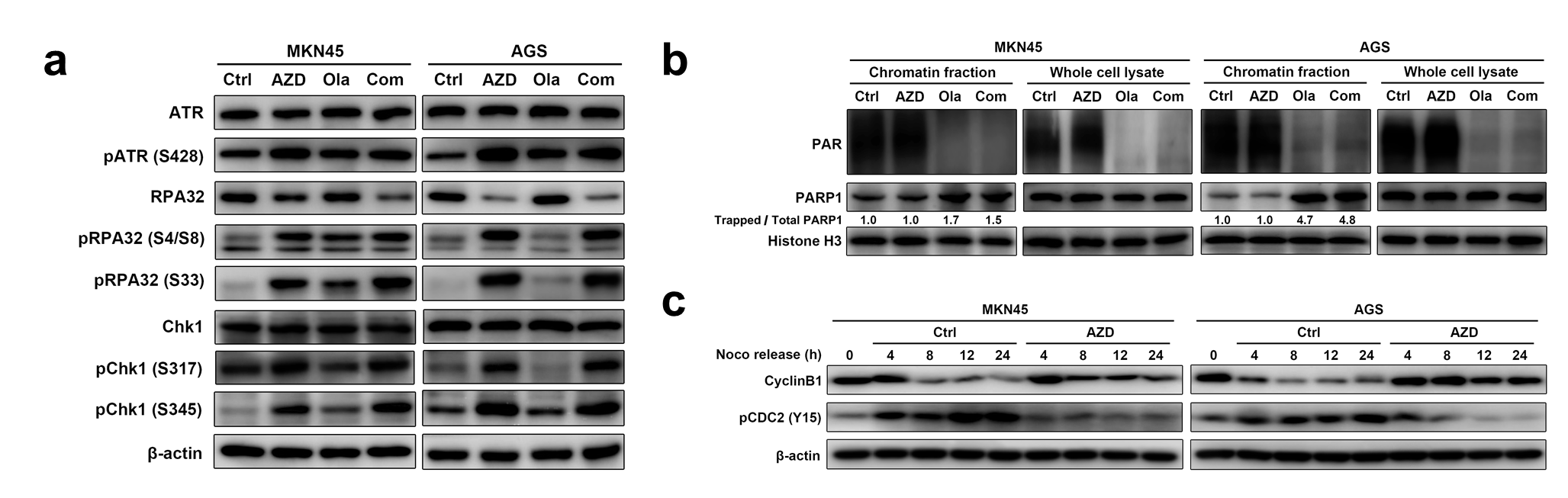

Supplement: Supplementary file 2 — Figure S2. Effects of AZD1775 plus olaparib on SSB accumulations, PARP trapping and prolonged mitosis in GC cells. (a-c) after drug treatment as indicated, proteins extracted from whole cell lysates or chromatin were probed with indicated antibodies. Trapped/total PARP1 indicated the ratios of PARP1 levels in chromatin to PARP1 levels in whole cell lysates which were then normalized to controls. Noco, Nocodazole. (TIF 647 kb) [file 13046_2018_790_MOESM2_ESM.tif]
